# Analysis of Corneal Spherical Aberrations in Cataract Patients with High Myopia

**DOI:** 10.1038/s41598-018-36539-1

**Published:** 2019-02-05

**Authors:** Min Zhang, Dongjin Qian, Qinghe Jing, Jiahui Chen, Yongxiang Jiang

**Affiliations:** 1 Department of Ophthalmology and Vision Science, Eye and ENT Hospital of Fudan University, Shanghai, China; 2 Key Laboratory of Myopia of State Health Ministry, and Key Laboratory of Visual Impairment and Restoration of Shanghai, Shanghai, China

## Abstract

To evaluate the corneal spherical aberrations in cataract patients with and without high myopia, we conducted a retrospective case series of 502 cataract eyes with high myopia and 1500 age-related cataract eyes and measure their corneal biometric data and axial length using Pentacam and IOLMaster. Both the anterior and total corneal primary spherical aberrations were lower in the high myopia group than that in the control group (0.317 ± 0.215 vs 0.338 ± 0.148 μm, *P* = 0.043; and 0.281 ± 0.207 vs 0.314 ± 0.153 μm, *P* < 0.001). The incidence of eyes with negative total corneal primary spherical aberration increased as axial length increased in the high myopia group, and the overall incidence was higher in the high myopia group than that in the control group (2.59% vs 1.47%). These were mainly contributed to the younger age of cataract patients with high myopia (55.76 ± 13.10 vs 60.18 ± 15.72 years, *P* < 0.001), along with the positive correlations between age and anterior and total corneal primary spherical aberrations. In clinical practice, an aspheric IOL with a low negative or zero primary spherical aberration is recommended for cataract patients with high myopia. Negative total ocular primary spherical aberrations resulting from aspheric IOL implantation should be avoided in extremely high myopic eyes.

## Introduction

Intraocular lenses (IOLs) were designed to add spherical refractive power to the ocular optical system from which the neutral lenses had been removed. Further refinements were made to mimic specific ocular properties, such as the development of aspheric IOLs to compensate for positive corneal spherical aberrations^1^. The reduction of ocular spherical aberrations can lead to a better retinal image and optimized visual performance, even when the best corrected visual acuity remains the same^2,3^.

The designs of aspheric IOLs are based on the average corneal spherical aberrations reported in large-scale clinical studies to achieve ideal outcomes for subjects with ocular aspherical aberrations. However, few studies have investigated the distribution of corneal spherical aberrations in cataract patients with high myopia, so the suitability of aspheric IOLs for these patients has not been demonstrated.

The global prevalence of high myopia was estimated to be 163 million individuals (2.7% of the world population and 11.6% of all myopia patients) in 2000 and was predicted to be 938 million (9.8% of the world population and 19.7% of all myopia patients) by 2050^4^. We have also identified an increasing trend in the proportion of subjects with high myopia cataract compared with age-related cataract in our clinical practice in recent years.

In general, cataract patients with high myopia are well educated and have high expectations of their postoperative subjective and objective visual performance (clearer, more comfortable, and longer lasting vision). Both patients and surgeons aspire not only to improvements in visual acuity and contrast sensitivity, but also to the relief and elimination of visual complaints such as starbursts and glare^5^.

Yu *et al*. compared the spherical aberrations after the implantation of aspheric and spherical IOLs in highly myopic subjects and found that aspheric IOLs were helpful^6^. However, the exact distribution of corneal spherical aberrations in highly myopic patients is still unknown. Considering the increasing prevalence of high myopia around the world and the high expectations of society overall, there is an obvious need for ophthalmologists to determine this distribution.

Consequently, in this study, we (1) determined the distributions of primary spherical aberrations in cataract patients with high myopia; (2) compared the corneal spherical aberrations in control cataract patients and cataract patients with high myopia; and (3) identified the main factors associated with spherical aberrations in highly myopic patients. We evaluated these factors and confirmed the effectiveness of aspheric IOL implantation during cataract surgery in patients with high myopia. Some suggestions are also made for the proper choice of aspheric IOLs.

## Methods

In this retrospective study, we recruited patients scheduled for cataract surgery from July 10 to December 21, 2017, at the Eye and ENT Hospital of Fudan University, Shanghai, China. The inclusion criteria were age-related or high myopia cataract and the ability to understand and sign the informed consent form. Corneal biometric data and axial length were determined in all the patients. Patients with a history of previous ocular trauma or surgery, diagnosed dry eye disease, corneal comorbidities, such as uveitis or glaucoma, fundus abnormalities, or diabetic retinopathy, or who had worn contact lenses within the previous 2 weeks, were excluded. The patients were divided into two groups according to their axial length (cutoff = 26 mm): cataract patients with high myopia (high myopia group) and cataract patients without high myopia (control group). This study was approved by the Human Research Ethics Committee of the Eye and ENT Hospital of Fudan University and adhered to the tenets of the Declaration of Helsinki. Written informed consent was obtained from each patient.

All data were collected in auto-mode, centered at corneal apex, with a rotating Scheimpflug camera (Pentacam; Oculus, Wetzlar, Germany) and partial coherence interferometry (IOLMaster; Carl Zeiss Meditec, Jena, Germany) by each of two skillful examiners to eliminate any latent examiner bias as far as possible. The examination was considered valid only if the quality expressed by the software was “OK”. Each patient’s last acceptable reading was used for the subsequent analysis. The measurements made at a pupil scan of 6 mm diameter included steep keratometric power, anterior and posterior corneal astigmatism, central corneal thickness (CCT), eccentricity (Ecc), Q value and the primary spherical aberration (Z 4 0) of the total cornea, anterior corneal surface, and posterior corneal surface. Conic coefficients were collected at a pupil scan of 8 mm diameter: Index of Surface Variance (ISV), Index of Vertical Asymmetry (IVA), Keratoconus Index(KI), Center Keratoconus Index (CKI), Index of Height Asymmetry (IHA), and Index of Height Decentration (IHD). The acquired corneal aberration data sets were expanded with normalized Zernike polynomials, with the magnitudes of their coefficients represented as the root mean square (in micrometers) and used to indicate wavefront aberrations. With-the-rule (WTR) astigmatism was defined as a cylindrical error for a steep corneal meridian of 90° ± 30° and against-the-rule (ATR) astigmatism was defined as a cylindrical error for a steep corneal meridian of 0° ± 30°. All other astigmatic readings outside these parameters were designated ‘oblique astigmatism’.

### Statistical analysis

To avoid any potential contralateral effect or sympathetic effect, we recruited and included in the statistical analysis only one eye of patients who were scheduled for cataract surgery. To analyze the incidence of negative spherical aberration of the total cornea or the anterior corneal surface, the high myopia patients were stratified into three categories according to their axial length: ≥26 and <28; ≥28 and <30; and ≥30 mm.

All continuous data are presented as means ± standard deviations (SD). Statistical analysis of the quantitative data was performed for all variables. The Kolmogorov–Smirnov test was used to assess the normality of the distributions of continuous data. An independent-samples *t* test was performed for all consecutive items and Pearson’s χ^2^ test and Fisher’s exact test were used to compare categorical items in the high myopia and control groups. The exact statistical contributions of explanatory variables, such as age, axial length, CCT, corneal astigmatism, and steep keratometric power, to the spherical aberrations were investigated with a multiple regression analysis with a backward selection technique. All data were analyzed with SPSS 23.0 (SPSS, IBM Corp., Armonk, NY, USA). A *P* value < 0.05 was considered to indicate statistical significance.

## Results

**Figure 1:**
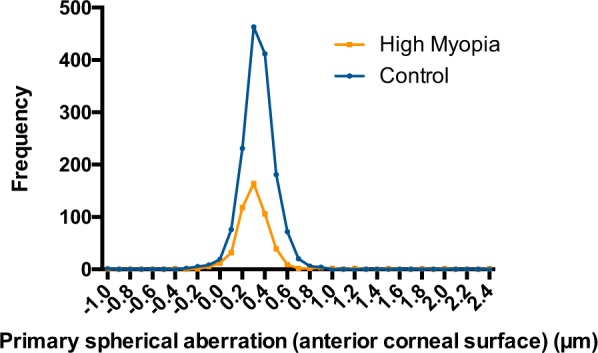
The distributions of anterior corneal primary spherical aberration in two groups. The ranges of anterior corneal primary spherical aberration were −1.009 to 0.918 μm in the control group and −0.400 to 2.421 μm in the high myopia group. Patients in the high myopia group had a lower value than those in the control group (0.317 ± 0.215 vs. 0.338 ± 0.148 μm, *P* = 0.043).

**Figure 2:**
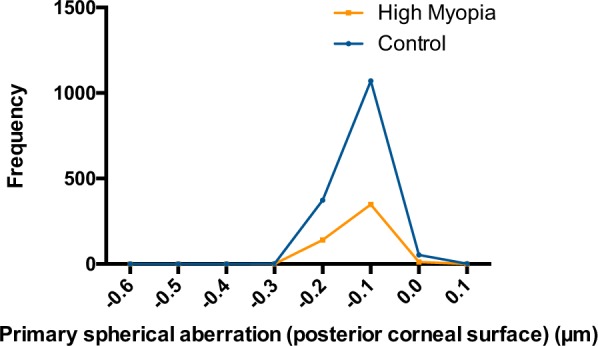
The distributions of posterior corneal primary spherical aberration in two groups. The ranges of posterior corneal primary spherical aberration were −0.328 to 0.097 μm in the control group and −0.645 to −0.017 μm in the high myopia group. Patients in the high myopia group had a slightly higher absolute value than those in the control group (−0.130 ± 0.044 vs. −0.123 ± 0.040 μm, *P* = 0.002).

**Figure 3:**
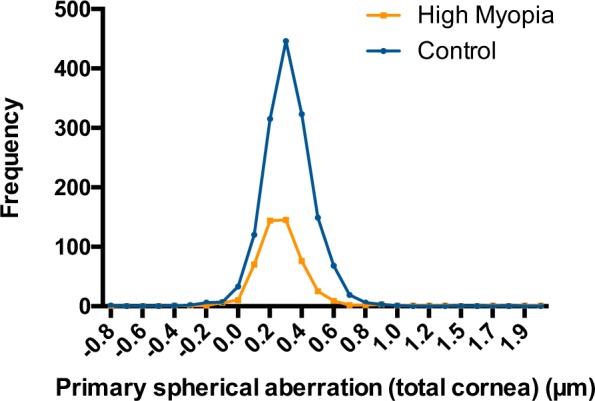
The distributions of total corneal primary spherical aberration in two groups. The ranges of anterior corneal primary spherical aberration were −0.777 to 0.981 μm in the control group and −0.389 to 1.969 μm in the high myopia group. Patients in the high myopia group had a lower value than those in the control group (0.281 ± 0.207 vs. 0.314 ± 0.153 μm, *P* < 0.001).

A total of 2002 eyes of 2002 patients were enrolled in this study: 502 eyes in the high myopia group and 1500 eyes in the control group. The demographic data, corneal biometric data, and axial lengths of these patients are listed in Table 1, with comparisons between the two groups. The distributions of primary spherical aberrations of the total cornea and the anterior and posterior corneal surfaces are presented in Figs 1–3. The results of the multiple linear regression analyses of the high myopia group, the control group, and the whole study population are presented in the Supplementary Material.

**Table 1:** Comparisons of demographic data, corneal biometric data, and axial lengths of patients in the control and high myopia groups.

Group		Control		High Myopia					Total	*P* value
Axial length (mm)		<26		>=26 & <28	>=28 & <30	>=30	Subtotal			
Eyes		1500		197	115	190	502		2002	—
Male/Female		589/911		—	—	—	214/288		803/1199	0.189**
Age (years)		60.18 ± 15.72		—	—	—	55.76 ± 13.10		59.07 ± 15.22	<0.001*
Astig CF (D)		0.831 ± 0.731		—	—	—	1.069 ± 0.716		0.890 ± 0.734	<0.001*
Axis CF	WTR	683	45.53%	108	55	85	248	49.40%	931	0.016**
	ATR	537	35.80%	58	31	56	145	28.88%	682	
	Oblique	280	18.67%	31	29	49	109	21.71%	389	
Astig CB (D)		0.282 ± 0.158		—	—	—	0.294 ± 0.165		0.285 ± 0.160	0.129*
Axis CB	WTR	1325	88.33%	180	93	162	435	86.65%	1760	0.500**
	ATR	127	3.20%	6	8	7	21	4.18%	69	
	Oblique	48	8.47%	11	14	21	46	9.16%	173	
Steep Km CF (D)		43.771 ± 1.564		—	—	—	43.007 ± 1.996		43.579 ± 1.714	<0.001*
Steep Km CB (mm)		−6.388 ± 0.263		—	—	—	−6.255 ± 0.293		−6.355 ± 0.277	<0.001*
CCT (mm)		539.77 ± 32.13		—	—	—	539.99 ± 33.43		539.89 ± 32.45	0.897*
Q value CF		−0.33 ± 0.18		—	—	—	−0.29 ± 0.30		−0.32 ± 0.22	0.001*
Ecc CF		0.50 ± 0.19		—	—	—	0.47 ± 0.31		0.49 ± 0.22	0.031*
Q value CB		−0.46 ± 0.46		—	—	—	−0.40 ± 0.18		−0.45 ± 0.41	0.006*
Ecc CB		0.60 ± 0.16		—	—	—	0.56 ± 0.19		0.59 ± 0.17	<0.001*
ISV		19.31 ± 11.58		—	—	—	20.27 ± 12.14		19.55 ± 11.73	0.114*
IVA		0.16 ± 0.10		—	—	—	0.16 ± 0.13		0.16 ± 0.11	0.571*
KI		1.02 ± 0.03		—	—	—	1.01 ± 0.04		1.02 ± 0.04	0.015*
CKI		1.00 ± 0.01		—	—	—	0.01 ± 0.01		1.00 ± 0.01	0.817*
IHA		5.81 ± 5.55		—	—	—	5.78 ± 5.10		5.80 ± 5.44	0.924*
IHD		0.01 ± 0.01		—	—	—	0.01 ± 0.01		0.01 ± 0.01	0.225*
Z 4 0 CF (μm)	Mean ± SD	0.338 ± 0.148		—	—	—	0.317 ± 0.215		0.333 ± 0.168	0.043*
	Positive	1476	98.40%	196	113	183	492	98.01%	1968	—
	Negative	24	1.60%	1	2	7	10	1.99%	34	—
Z 4 0 CB (μm)	Mean ± SD	−0.123 ± 0.040		—	—	—	−0.130 ± 0.044		−0.125 ± 0.041	0.002*
	Positive	6	0.40%	0	0	0	0	0.00%	6	—
	Negative	1494	99.60%	197	115	190	502	100%	1996	—
Z 4 0 Cornea (μm)	Mean ± SD	0.314 ± 0.153		—	—	—	0.281 ± 0.207		0.306 ± 0.168	<0.001*
	Positive	1478	98.53%	197	113	179	489	97.41%	1967	—
	Negative	22	1.47%	0	2	11	13	2.59%	35	—

SD = standard deviation; CCT = central corneal thickness; D = diopter; CF = front/anterior corneal surface; CB = back/posterior corneal surface; Cornea = total corneal aberrations; steep Km = steep meridian keratometric power; Astig = astigmatism; Axis = the axis of a steep meridian; Ecc = eccentricity; ISV = Index of Surface Variance; IVA = Index of Vertical Asymmetry; KI = Keratoconus Index; CKI = Center Keratoconus Index; IHA = Index of Height Asymmetry; IHD = Index of Height Decentration; Z 4 0 = primary spherical aberration.

*P values determined with independent-samples *t* test.

**P values determined with Pearson’s chi-square test.

The average age of patients in the high myopia group was lower than that in the control group (55.76 ± 13.10 vs 60.18 ± 15.72 years, *P* < 0.001). In the multiple linear regression analysis of all enrolled patients, age correlated positively with primary spherical aberration of the total cornea and the anterior and posterior corneal surfaces (all three, *P* < 0.001).

Although the criterion used to divide the groups was axial length, axial length itself showed no or only a weak correlation with the spherical aberrations, especially the primary spherical aberration.

Anterior corneal astigmatism contributed to the primary spherical aberration in the control group (negatively for the total cornea and the anterior corneal surface and positively for the posterior corneal surface, all *P* < 0.001), but not in the high myopia group. The posterior corneal astigmatism in the high myopia group was similar to that in the control group (*P* = 0.129), and it was almost not identified as a significant factor in the multiple linear regression analyses of spherical aberrations (only in the analyses of anterior and posterior corneal primary spherical aberration with *P* = 0.054 and *P* = 0.002, respectively). A slightly lower proportion of ATR astigmatism was detected in the high myopia group than that in the control group (28.88% vs 35.80%, *P* = 0.016). The steep meridian keratometric powers of the both anterior and posterior corneal surfaces correlated negatively with the spherical aberrations. No statistically significant difference was identified in CCT between the two groups (539.99 ± 33.43 in the control group vs 539.77 ± 32.13 mm in the high myopia group, *P* = 0.897). Both Ecc and Q value of anterior and posterior corneal surface differed between two groups, while among all conic coefficients only KI differed (1.02 ± 0.03 in the control group vs 1.01 ± 0.04 in the high myopia group, *P* = 0.015).

Both the anterior and total corneal primary spherical aberrations were greater in the control group than that in the high myopia group (0.338 ± 0.148 vs 0.317 ± 0.215 μm, *P* = 0.043; and 0.314 ± 0.153 vs 0.281 ± 0.207 μm, *P* < 0.001). The incidence of eyes with negative total corneal primary spherical aberration increased as the axial length increased in the high myopia group (0.00%, 1.74%, and 5.79% in the three axial length categories, respectively), and the total incidence was higher in the high myopia group than that in the control group (2.59% vs 1.47%). Similar outcomes were detected for anterior corneal primary spherical aberrations: the incidence in the three axial length categories (≥26 and <28; ≥28 and <30; and ≥30 mm) were 0.51%, 1.74%, and 3.68%, respectively, and the total incidence was higher in the high myopia group (1.99%) than that in the control group (1.60%).

## Discussion

Spherical aberrations have compelling and marked effects on optical quality, but among them, primary spherical aberrations can be compensated and partly adjusted with aspheric IOLs, improving visual performance^6^. Therefore, spherical aberrations have attracted our attention and that of many other ophthalmologists.

Recent researches into corneal spherical aberrations are presented in Table 2. Our findings for the primary spherical aberration in the control group are consistent with the findings of other researches in cataract patients.

**Table 2:** Previous results for the distributions of corneal spherical aberrations.

Author	Year of publication	Race	Identification		Number of patients	Number of eyes	Male/female	Age (years)		Axial length (mm)	Divice	Diameter of analyzed area (mm)	Z 4 0 (μm)		
								Range	Mean ± SD				Total cornea	Anterior surface	Posterior surface
Al-Sayyari *et al*.^7^	2014	Saudi	normal		185	300	97/88	15 to 85	—	—	Pentacam HR	6	0.252 ± 0.1154	—	—
Beiko *et al*.^8^	2007	German	normal	OD	301	301	111/190	—	64.0 ± 16.1	—	Easygraph (Oculus)	6	0.273 ± 0.095	—	—
				OS		301				—		6	0.275 ± 0.097	—	—
Al-Sayyari *et al*.^1^	2014	Saudi	cataract		53	45	25/20	45 to 90	—	—	Pentacam HR	6	0.3354 ± 0.1965	—	—
Yuan *et al*.^9^	2014	Chinese	cataract	Group1	153	922	—	60 to 70	73.5 ± 6.8	—	Pentacam HR	—	—	0.361 ± 0.122	−0.122 ± 0.035
				Group2	251		—	70 to 80		—		—	—	0.401 ± 0.139	−0.105 ± 0.040
				Group3	100		—	80 to 90		—		—	—	0.440 ± 0.145	−0.090 ± 0.043
Li *et al*.^10^	2012	Chinese	catarract		93	155	45/48	50 to 89	—	—	Pentacam HR	6	0.294 ± 0.138	—	—
Shimozono *et al*.^11^	2010	Japanese	cataract		168	257	—	—	—	—	Wavefront analyzer (KR9000PW, Topcon)	6	0.203 ± 0.100	—	—
Negishi *et al*.^12^	2010	Japanese	cataract		29	37	11/18	58 to 83	70.8 ± 7.4	—	OPD-Scan	6	0.27 ± 0.23	—	—
Yu *et al*.^6^	2009	Chinese	cataract, high myopia	aspherical group	31	22	—	—	54.550 ± 6.061	30.210 ± 1.593	WASCA wavefront analyser (Carl Zeiss, Oberkochen, Germany)	6	0.30 ± 0.11	—	—
				spherical group		23	—	—	52.610 ± 5.525	30.960 ± 2.045		6	0.29 ± 0.13	—	—
Tong *et al*.^13^	2007	Chinese	cataract		144	188	—	—	—	—	Ray-training calculation programs	—	—	0.231 ± 0.092	—
Guirao *et al*.^14^	2004	Spainish	cataract		—	70		32 to 89	70 ± 12	23.6 ± 2.1	Corneal topographer	6	0.32 ± 0.12	—	—
de Sanctis *et al*.^15^	2014	Italian	cataract		149	149	57/92	—	71.73 ± 9.12	—	Pentacam HR	6	0.328 ± 0.132	0.353 ± 0.132	−0.121 ± 0.034
Zhang *et al*.^16^	2017	Chinese	scheduled for SMILE		134	70	40/30	—	22.16 ± 3.87	—	Pentacam HR	6	0.21 ± 0.08	0.25 ± 0.07	−0.15 ± 0.02
			scheduled for FS-SMILE			64	34/30	—	23.22 ± 3.62	—		6	0.18 ± 0.07	0.23 ± 0.07	−0.16 ± 0.02
Kingston *et al*.^17^	2013	multiple sites in Asia, North America, Europe, and Australia	Scheduled for LASIK		—	1124	—	19 to 45	31.8	—	Pentacam HR	6	0.18	—	—
Ahn *et al*.^18^	2013	Korean	Schduled for PTK		25	26	10/15	—	53 ± 16.8	—	Myadriatics (Mydrin-p; Santen, Osaka, Japan)	more than 6 mm	0.291 ± 0.094	—	—
			Scheduled for conventional PTK		14	26	1/13	—	27.3 ± 3.2	—			0.270 ± 0.056	—	—
			Scheduled for wave-front guided LASEK		17	34	5/12	—	31 ± 4.7	—			0.286 ± 0.083	—	—
Bottos *et al*.^19^	2011	American	Scheduled for refractive surgery	myopia	—	177	—	22 to 63	35 ± 8	—	Pentacam HR	6	0.21 ± 0.08	0.27 ± 0.07	−0.17 ± 0.03
				hyperopia	—	32	—	33 to 71	55 ± 11	—		6	0.36 ± 0.11	0.38 ± 0.10	−0.14 ± 0.04

Methods: PubMed was searched with key word “spherical aberration” and (“cornea” OR “corneal”) in all files to April, 3, 2018.

Inclusion criteria: recruited patients were normal people, cataract patients or those scheduled for corneal refractive surgery, with no history of ocular trauma, surgery, or corneal comorbidity (such as glaucoma). Preoperative corneal spherical aberrations were measured in all patients, and no contact lens wear was permitted in the 2 weeks before measurement.

In addition, not included in this table were studies in which the recruited patients were divided into several subgroups based on their spherical aberrations, and the spherical aberration values of each subgroup were reported rather than the values of all the patients.

The design of aspheric IOLs was based on large-scale studies of corneal spherical aberrations in the normal population, and they are only known to be appropriate for eyes with age-related cataract^2^. At present, the widely used aspheric IOLs have a primary spherical aberration of −0.27 μm or −0.20 μm, and conventional zero spherical aberration IOLs are selected for patients with low or negative corneal spherical aberrations^1^. However, in China, these choices are sometimes made regardless of the patient’s corneal spherical aberrations. Spherical aberrations of the anterior corneal surface and total corneal primary spherical aberrations were both smaller in the high myopia group than that in the control group (0.317 ± 0.215 vs 0.338 ± 0.148 μm, *P* = 0.043; and 0.281 ± 0.207 vs 0.314 ± 0.153 μm, *P* < 0.001). Therefore, it seems inappropriate to recommend the same aspheric IOLs for patients with age-related cataract and for those with high myopia.

At the outset of aspheric IOL development, the Tecnis Z9000 (Abbott Medical Optics, Inc.), with a primary spherical aberration of −0.27 μm, was deliberately designed to negate all the positive primary spherical aberrations of the cornea, and it effectively reduces ocular spherical aberrations after implantation^7^. Though the correction of spherical aberrations makes improvement in image quality, residual spherical aberrations are not devoid of any merit. Appropriate reservation of spherical aberrations can polish up the depth of focus and distance-corrected near and intermediate visual acuity to some extent^8,9^. Now the personalized correction of primary spherical aberrations is performed in cataract surgery, and a postoperative mean residual spherical aberration of approximately 0.1 μm results in better contrast sensitivity and therefore better visual quality^7,10,11^. In the present study, the primary spherical aberration of the total corneal surface in the high myopia group had a mean value of 0.281 μm. Using aspheric IOLs with a small primary spherical aberration compensation, such as −0.20 μm, we can achieve a residual primary spherical aberration of no less than 0.081 μm, very close to 0.1 μm, which provides the best contrast sensitivity and ideal visual outcomes for these patients.

One noteworthy finding was the incidence of negative corneal primary spherical aberrations. A slight increase in the number of eyes with negative total corneal primary spherical aberration was detected as the axial length increased in the high myopia group (0.00%, 1.74%, and 5.79% in three axial length categories, respectively), and the incidence in the whole group was higher than that in the controls (1.47% in the control group vs 2.59% in the high myopia group).

The increasing tendency for negative corneal primary spherical aberrations to occur in patients in the ≥26 and <28 mm group, ≥28 and <30 mm group and ≥30 mm group can be explained by the decreasing corneal primary spherical aberrations with increasing axial length in the high myopia group, which itself may be attributable to the lower age of the subjects in the high myopia group.

It is well recognized that high myopia is a risk factor for cataract and that highly myopic patients tend to suffer cataract at a younger age than the normal population^12^. Our results are consistent with those of previous studies, showing a lower average age in the high myopia group than that in the control group (55.76 ± 13.10 vs 60.18 ± 15.72 years, *P* < 0.001). Age is also considered to correlate positively with spherical aberrations^13^. This relationship is also supported by previous results (Table 2). Therefore, it is not unusual for corneal primary spherical aberrations to be less common in the younger group (high myopia group), which was strongly confirmed with the multiple linear regression analyses in this study (the standardized coefficients for age were all >0.2, with *P* < 0.001, for the total, anterior, and posterior corneal spherical aberrations).

With adjustment for age in the regression analyses, axial length itself, the criterion upon which the groups were divided, did not correlate statistically with anterior or total corneal primary spherical aberration. This differs from the low negative correlation between corneal primary spherical aberration and axial length demonstrated by Al-Sayyari *et al*.^1^. However, several theories can at least partly explain the smaller primary spherical aberrations in the high myopia group in this study.

Animal experiments and clinical studies have shown that subjects with myopia tend to have a more negative spherical aberration with accommodation and positive spherical aberration slowed the axial growth of the eye^14–18^. Thus, the implantation of aspheric IOLs in cataract patients with high myopia must be undertaken with caution. A preoperative examination of corneal aberrations should be performed with care, and prudent decisions should be made on the aspheric IOL selected. It is unclear whether postoperative negative total ocular primary spherical aberrations arising from overcompensation will increase the axial length and exacerbate pathological myopia. To avoid this potential risk, particular attention must be paid to these patients and individualized spherical aberration adjustments are warranted. If this is not possible, zero-spherical-aberration IOLs might be the best second choice.

However, it is really difficult for us to pick up and hardly practical for ophthalmologists to employ a cutoff age under which patients are more suitable for the aspheric IOLs with a low negative or zero primary spherical aberration if no personalized primary spherical reduction is achieved in the cataract surgery. “High myopia” is widely recognized and can be easily detected. Ophthalmologists would always pay serious attention to the design of these patients’ IOLs due to their complex ocular condition. These process would be easier when keep the following in mind: An aspheric IOL with a low negative or zero primary spherical aberration is recommended for cataract patients with high myopia. Negative total ocular primary spherical aberrations resulting from aspheric IOL implantation should be avoided in extremely high myopic eyes. These two were the keys of our investigations.

There were some limitations in this study. First, the use of Scheimpflug analysis alone may have limited the acuity of the corneal biometrics in the high myopia group. Although previous studies have reported the repeatability and reproducibility of measurements of the anterior segment made with the Scheimpflug instrument in normal eyes, the poor preoperative fixation stability in cataract patients with long axial lengths could lead to fluctuations in the data^19,20^. Therefore, more stable corneal topography is required. Second, we did not measure ocular surface dryness. Despite the exclusion of all eyes with a diagnosis of dry eye, different tear film conditions still affect the measurement of aberrations^21^. Last but not least, we did not analyze the spherical aberrations after aspheric IOL implantation. Had we done so, we could have evaluated the validity of our recommendation. However, too many patients were recruited to be followed up over a short period. Testing and confirmation of our recommendation will be the focus of our future work.

## Conclusions

Cataract patients with high myopia had younger age and smaller corneal primary spherical aberrations and an increased proportion of negative corneal primary spherical aberrations than age-related cataract patients did. The incidence of negative corneal primary spherical aberrations increased slightly as the axial length increased, although the exact number of these patients was small. These were mainly contributed to the positive correlations between age and anterior and total corneal primary spherical aberrations. In clinical practice, an aspheric IOL with a low negative primary spherical aberration is recommended for cataract patients with high myopia. Cataract patients with extremely high myopia should be considered seriously. Negative total ocular spherical aberrations resulting from aspheric IOL implantation in extremely high myopic eyes should be avoided.

## Electronic supplementary material

Results of multiple linear regression analyses
