# Supplementary material for: Analysis of Corneal Spherical Aberrations in Cataract Patients with High Myopia
Source: Sci Rep. 2019 Feb 5;9:1420. doi: 10.1038/s41598-018-36539-1 (PMC6363797; doi:10.1038/s41598-018-36539-1)
Supplement: Supplementary file 1 — Results of multiple linear regression analyses [file 41598_2018_36539_MOESM1_ESM.docx]

**Analysis of Corneal Spherical Aberrations in Cataract Patients with High Myopia**

**Running Head:** Corneal Spherical Aberrations in High Myopes

**Authors:** Min Zhang^1,2^**;** Dongjin Qian^1,2^, PhD; Qinghe Jing^1,2^, MM; Jiahui Chen^1,2^, BM; Yongxiang Jiang^1,2*^, MD, PhD

**Affiliations:**

^1^Department of Ophthalmology and Vision Science, Eye and ENT Hospital of Fudan University, Shanghai, China

^2^Key Laboratory of Myopia of State Health Ministry, and Key Laboratory of Visual Impairment and Restoration of Shanghai, Shanghai, China

***Corresponding author:** Yongxiang Jiang, Department of Ophthalmology, Eye and ENT Hospital of Fudan University, 83 Fenyang Rd., Shanghai, China, 200031

Tel: +86 021 64377134; Fax: +86 021 64377151; E-mail: yongxiang_jiang@163.com

**Acknowledgements**

This study was funded by the National Natural Science United Foundation of China (grant no. U1503124) and the National Natural Science Foundation of China (grant no. 81770908).

**Keywords:** high myopia; spherical aberration; cataract; IOL; associated factor

**Financial Disclosure:** None of the authors has any conflict of interest to disclose. The authors have no propriety or commercial interest in any product discussed in this article.

Supplementary material: Results of multiple linear regression analyses

|  | | Age | | | Axial length | | | Km F (D) | | | Astig F | | | Km B | | | Astig B | | | CCT | | |
| --- | --- | --- | --- | --- | --- | --- | --- | --- | --- | --- | --- | --- | --- | --- | --- | --- | --- | --- | --- | --- | --- | --- |
|  |  | b | Beta | *P* value | b | Beta | *P* value | b | Beta | *P* value | b | Beta | *P* value | b | Beta | *P* value | b | Beta | *P* value | b | Beta | P value |
| Z 4 0 CF | Total | 0.003 | 0.306 | <0.001 | - | - | - | -0.051 | -0.522 | <0.001 | -0.015 | -0.066 | 0.001 | -0.342 | -0.565 | <0.001 | - | - | - | -0.001 | -0.167 | <0.001 |
|  | Control | 0.003 | 0.345 | <0.001 | - | - | - | -0.018 | -0.187 | <0.001 | -0.024 | -0.120 | <0.001 | -0.183 | -0.325 | <0.001 | - | - | - | 0.000 | -0.092 | <0.001 |
|  | High myopia | 0.004 | 0.259 | <0.001 | - | - | - | -0.081 | -0.755 | <0.001 | - | - | - | -0.452 | -0.615 | <0.001 | 0.094 | 0.073 | 0.054 | -0.001 | -0.187 | <0.001 |
| Z 4 0 CB | Total | 0.001 | 0.365 | <0.001 | -0.001 | -0.108 | <0.001 | -0.008 | -0.325 | <0.001 | 0.003 | 0.056 | 0.007 | - | - | - | - | - | - | 8.837E-05 | 0.071 | <0.001 |
|  | Control | 0.001 | 0.409 | <0.001 | -0.002 | -0.067 | 0.010 | -0.011 | -0.445 | <0.001 | 0.006 | 0.114 | <0.001 | -0.014 | -0.092 | 0.042 | - | - | - | - | - | - |
|  | High myopia | 0.001 | 0.207 | <0.001 | - | - | - | -0.005 | -0.242 | <0.001 | - | - | - | - | - | - | -0.036 | -0.137 | 0.002 | 0.000 | 0.162 | <0.001 |
| Z 4 0 Cornea | Total | 0.004 | 0.386 | <0.001 | - | - | - | -0.056 | -0.547 | <0.001 | 0.013 | 0.055 | 0.004 | -0.381 | -0.627 | <0.001 | - | - | - | -0.001 | -0.153 | <0.001 |
|  | Control | 0.004 | 0.432 | <0.001 | - | - | - | -0.025 | -0.255 | <0.001 | -0.018 | -0.087 | <0.001 | -0.226 | -0.390 | <0.001 | - | - | - | 0.000 | -0.086 | <0.001 |
|  | High myopia | 0.005 | 0.295 | <0.001 | - | - | - | -0.086 | -0.825 | <0.001 | - | - | - | -0.501 | -0.708 | <0.001 | - | - | - | -0.001 | -0.160 | <0.001 |

b = coefficient; Beta = standardized coefficient; CCT = central corneal thickness; D = diopter; CF = front/anterior corneal surface; CB = back/posterior corneal surface; Cornea = total corneal aberrations; Km = steep meridian keratometric power; Astig = astigmatism; Z 4 0 = primary spherical aberration.

Sign “–” indicates no statistical significance (*P* ≥ 0.05) in the multiple linear regression analyses.
